# Simultaneous population genomics of hosts and their parasites with selective whole genome amplification

**DOI:** 10.1186/s13071-025-07087-1

**Published:** 2025-11-05

**Authors:** Vincenzo A. Ellis, Angela Theodosopoulos, Ishika Sharma, Amélie Bardil, Martin Stjernman, Olof Hellgren

**Affiliations:** 1 Department of Entomology and Wildlife Ecology, University of Delaware, Newark, DE USA; 2 Department of Biology, Lund University, Lund, Sweden; 3 CEFE, CNRS, EPHE, University Montpellier, Montpellier, IRD France

**Keywords:** Avian malaria, Coevolution, Host genomics, Parasite evolution, Pathogen genomics

## Abstract

**Background:**

Generating parasite genomes is challenging when little of the DNA in infected host tissue is from the parasite. We used selective whole genome amplification (SWGA) to generate genomic data from wildlife samples of the avian haemosporidian *Haemoproteus majoris* (lineage PARUS1) and its host, the blue tit (*Cyanistes caeruleus*).

**Methods:**

We used SWGA to amplify the parasite DNA in nine avian blood samples collected between 1996 and 2021, and subsequently performed short-read sequencing and bioinformatically separated the host and parasite reads in each sample.

**Results:**

SWGA increased the percentage of parasite reads significantly. Sequencing to a depth of about 56 million reads (forward and reverse) per sample resulted on average (± standard error [SE]) in 11.3X ± 1.85 for the host genome and 1.17X ± 0.446 mean depth of coverage for the host and parasite, respectively, after SWGA. Furthermore, about 74% of the host genome (genome size approx. 1.2 Gb) and 33% of the parasite genome (approx. 23.9 Mb) had at least 1X coverage on average; two samples had 1X coverage of approximately 60% of the parasite genome. Parasite sequencing success was positively correlated with parasitemia. When comparing the parasite sequences in the four best samples, we identified 9895 sites (minimum 5X coverage) that varied among the infections. When filtering the full dataset to at least six samples per variant, we identified 14,512,339 and 7068 sites that varied among samples in the host and parasite populations, respectively, revealing variation among samples and years.

**Conclusions:**

SWGA facilitates dual host-parasite population genomics in this system and will greatly expand our understanding of host-parasite interactions over space and time.

**Graphical Abstract:**

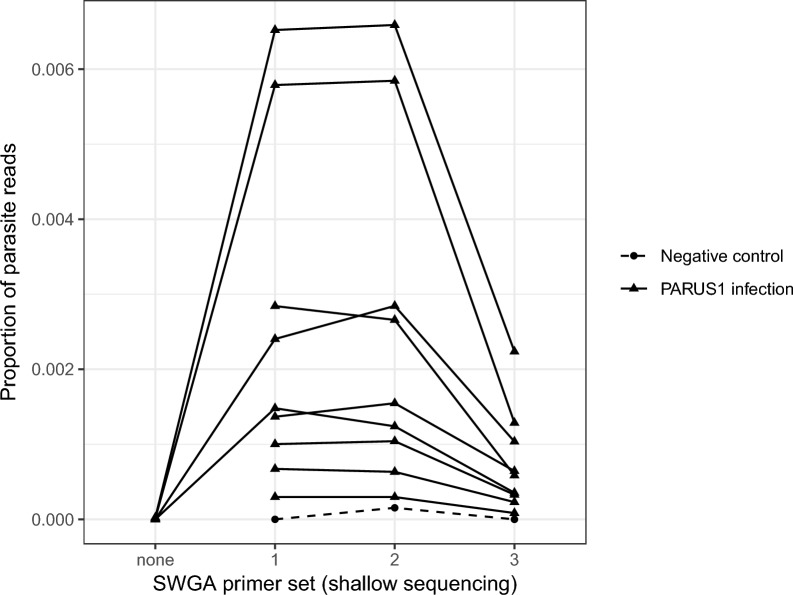

**Supplementary Information:**

The online version contains supplementary material available at 10.1186/s13071-025-07087-1.

## Background

Reciprocal selective pressures result in coevolutionary adaptations between hosts and parasites [1]. However, understanding the genetic underpinnings of host–parasite interactions and indeed documenting both host and parasite population genetics simultaneously are made challenging by the difficulty of sequencing parasite DNA in the presence of abundant host DNA. This problem is even more pronounced when working with wildlife, in which parasitemia (i.e. the number of parasites infecting an individual host) is typically lower than the level that can be obtained in controlled experimental infections in the laboratory. Selective whole genome amplification (SWGA) of parasites and pathogens has become an important tool for facilitating parasite whole genome sequencing [2]. Importantly, host DNA is still sequenced after SWGA (with coverage spanning the genome, since host DNA is not removed), providing an opportunity to leverage sequence reads of both the host and parasite to conduct host–parasite population genetic studies. Such studies can begin to address the genetic mechanisms underpinning host–parasite coevolution [3].

Avian haemosporidians (also known as avian malaria parasites) are a model system for studying host–parasite interactions and coevolution [4]. Avian haemosporidians are dipteran-transmitted protozoan parasites that infect birds globally [5, 6]. Samples of blood from an infected bird have both bird DNA and trace amounts of parasite DNA. However, because (i) avian red blood cells are nucleated and contain DNA; (ii) bird genomes are much larger than the parasite genomes [7] and are diploid whereas parasites in bird blood are haploid; and (iii) typically < 1% of bird red blood cells are infected in natural infections and consequently there is far more bird DNA than parasite DNA in infected wild birds [8]. Indeed, for the majority of samples obtained from the wild, less than approximately 1/18,000 of the genetic material sequenced would be expected to come from the parasite. While deep sequencing of a single infected host individual with unusually high parasitemia might be feasible for generating a single low-coverage parasite genome, it is not a feasible strategy for larger population-level studies. Several avian haemosporidian genomes [7, 9] and transcriptomes [10–15] have been sequenced, and techniques such as sequence capture have been used to obtain reduced representation genomic data [16–19] that can be used for population genomics and phylogenomics. However, all of these studies have either required experimental infections to generate high parasitemia levels or significant laboratory resources. Therefore, these techniques are suboptimal for large host–parasite population studies or host-by-parasite genotype association studies that require large sample sizes [20].

The avian haemosporidian system (and similar host parasite systems) could benefit from the development of SWGA. SWGA involves using primers that bind more frequently to the target genome (i.e. the parasite) than to the background genome (i.e. the host), with amplifications conducted isothermally using the phi29 enzyme. This technique has been used to study a variety of parasites, including human [21–24] and other primate [25, 26] malaria parasites, which are related to avian haemosporidians [7, 9]. However, humans do not have nucleated red blood cells, so the proportion of parasite to host DNA is less extreme than in the case of avian haemosporidians. Consequently the degree to which SWGA can improve avian haemosporidian genome sequencing success has remained unclear.

In the study reported here we conducted SWGA on infections of the avian haemosporidian *Haemoproteus majoris* (lineage PARUS1) in its host, the blue tit (*Cyanistes caeruleus*). *Haemoproteus majoris* is one of the most common *Haemoproteus* parasites transmitted to passerine birds in the eastern and western Palearctic [6] where it can infect several species in a bird community with prevalence in some species exceeding 50% [27]. In this study we used samples collected between 1996 and 2021 at the Stensoffa Field Station in southern Sweden. We identified primers that effectively amplify the genome of PARUS1 in the presence of high amounts of host DNA and demonstrate a bioinformatic approach to conducting population genomics of parasites and their hosts simultaneously.

## Methods

### Samples

Small blood samples were collected in SET buffer from the brachial or jugular vein of blue tits (*C. caeruleus*) at the Stensoffa Field Station (55.688 latitude, 13.480 longitude [decimal degrees]) during the bird breeding season between 1996 and 2021. DNA was extracted and tested for avian haemosporidians using standard protocols [28, 29]. Individuals found to be infected with the parasite *H. majoris* (lineage: PARUS1) through Sanger sequencing were selected for this study. Specifically, we selected three samples from 1996, three samples from 2008 and three samples from 2021 (i.e. 9 samples total). We also quantified the parasitemia of five of the samples using microscopy by counting the number of parasites (gametocytes) per 10,000 avian red blood cells. Sample details (including age and sex of the birds) can be found in Additional file 1: Table S1.

### Selective whole genome amplification

We designed primers that would selectively amplify the DNA of the target parasite in the presence of the DNA of its avian host using swga2.0 software [30]. The genome of the *H. majoris* lineage WW2 (ENA accession no.: GCA_965637345.1) and the genome of the blue tit (NCBI accession no.: GCA_030015615.1) were used in this analysis. The software produced 10 best scoring primer sets, from which we chose three to test (phosphorothioate bond modifications noted with *; these are necessary when performing amplifications with the phi29 enzyme), as follows:

- Primer set 1: [AAAAAATCA*A*A, AAAGAAACA*A*A, AAATGAAA*C*T, AATAAAATA*T*T];
- Primer set 2: [AAAAAATCA*A*A, AAAGAAACA*A*A, AAATGAAA*C*T, AAGAGATA*A*A, AATAAAATA*T*T];
- Primer Set 3: [AAAAAATCA*A*A, AAGAGATA*A*A, AATAAAATA*T*T, ATACATT*A*C, GAAAAAGT*G*A, TTGTTAA*G*T].

We conducted SWGA using the EquiPhi29 kit (Thermo Fisher Scientific, Waltham, MA, USA). The procedure occurs in two steps: (i) denaturation of the DNA and (ii) isothermal amplification. We diluted DNA samples to 25 ng/μl before starting. We ordered primers at a concentration of 200 μM in water and then made an equal volume (and therefore equal concentration) mixture of all primers in each set; thus the “primer set mix” was also at a concentration of 200 μM. For each sample, we mixed 2 μl of diluted DNA with 2.5 μl of the primer set mix and 0.5 μl of 10× EquiPhi29 Reaction Buffer over ice. To denature the DNA, we put the reaction mixtures into a MiniAmp thermocycler (Thermo Fisher Scientific) at 95 °C for 3 min with the machine’s hot lid turned on and then immediately put the reaction mixtures on ice for approximately 10 min. At the same time, we made the amplification master mix on ice: for each sample, we mixed 1.5 μl of 10× EquiPhi29 Reaction Buffer, 0.2 μl DTT (110 mM), 2 μl of dNTP mix (10 mM each; Thermo Fisher Scientific), 1 μl EquiPhi29 DNA polymerase (10 U/μl), 1 μl of pyrophosphotase (0.1 U/μl; Thermo Fisher Scientific) and 9.3 μl of ultrapure water. We then pipetted the resulting 15-μl volume of master mix directly into the 5-μl volume of denatured DNA and primer mix (still on ice), mixing briefly by pipetting up and down. Finally, we put the total volume of mix (20 μl) that included all reaction components and denatured DNA on the thermocycler at 45 °C for 3 h (isothermal amplification), followed by 65 °C for 10 min (enzyme deactivation); the hot lid was turned on for this step as well. We included a negative control (water) in each amplification and visualized the amplifications by running the amplified DNA in a 1.5% agarose gel for 1.5 h at 80 V, following which the DNA was stained with GelRed Nucleic Acid Gel Stain (Biotium, Fremont, CA, USA) and visualized with the GelDoc Go Imaging System (Bio-Rad Laboratories, Hercules, CA, USA).

The nine samples and negative control were amplified with each of the three primer sets. All samples were amplified in duplicate and combined prior to electrophoresis, library building and sequencing (i.e. each sample was amplified twice with each primer set and then the duplicate amplifications made with a single primer set were combined; different primer sets were not mixed together prior to library building).

### DNA sequencing

The amplified samples including the negative controls (*n* = 30) and four unamplified samples (one from 1996 [1EE56066]; one from 2008 [1EP50341]; two from 2021 [1HA37672 and 1EZ96477]) were brought to the University of Delaware DNA Sequencing and Genotyping Center (Newark, DE, USA) where they were purified with AMPure XP beads (Beckman Coulter, Brea, CA, USA) using a 1.5× ratio of beads to sample. Next, Illumina libraries were generated from the samples with the Illumina DNA (M) library preparation kit (Illumina, Inc., San Diego, CA, USA) following the manufacturer’s protocol and using 10 ng of purified DNA. The 27 amplified samples (9 for each primer set) were pooled in equimolar ratios and the three amplified negative controls (1 for each primer set) were added to the pool at a lower concentration. The pool was then sequenced on an Illumina MiSeq Sequencing System (paired end) using a V2 300 cycle sequencing kit (Illumina Inc.); we refer to this as the “shallow sequencing.” The nine samples amplified with primer set 2 were then separately pooled in equimolar ratios and sequenced on an Illumina NextSeq 2000 short-read sequencer (paired end) using a P2 300 cycle sequencing kit; we refer to this as the “deep sequencing.” Finally, the libraries built from the four unamplified samples were pooled in equimolar ratios and sequenced as part of a separate MiSeq run (paired end) using a Nano 500 cycle kit (Illumina, Inc.). Raw sequence reads are available on GenBank (BioProject ID: PRJNA1249433).

### Bioinformatics

We conducted our bioinformatic analyses on the BIOMIX high performance computational hardware (HPC) at the University of Delaware. Briefly, we removed adaptors and quality trimmed reads with trim galore v.0.6.6 (https://github.com/FelixKrueger/TrimGalore) and then mapped the reads from each sample to the blue tit genome (genome size: 1,201,937,744 bp) using BWA MEM v.0.7.17 algorithm [31]. We used samtools v.1.19.2 [32] to convert the resulting sam files to bam files and calculate mapping statistics. Next, we used samtools to extract unmapped reads from the bam files and convert the file format of those reads to interleaved fastq; this served to remove blue tit reads from the files. We mapped the host-removed reads to the WW2 reference genome (genome size: 23,881,874 bp) using BWA MEM (flag “-p” was used to accommodate the interleaved fastq file) and converted the sam files to bam and calculated mapping statistics using samtools as before; detailed mapping statistics are reported in Additional file 1: Table S2. For the deep sequencing samples, we used samtools to sort the bam files and Picard v.3.1.1 (https://github.com/broadinstitute/picard) to mark duplicates and add read groups to the bam files. We then indexed the duplicate marked and sorted bam files with samtools and proceeded to call variants from those files. To call variants, we first indexed the host and parasite reference genomes with samtools and created dictionary files with Picard. Then, we called variants (single nucleotide polymorphisms [SNPs] and insertions/deletions [indels]) with HaplotypeCaller in GATK v.4.3.0 [33, 34]; we used the flag “-ploidy 2” for host bam files and “-ploidy 1” for the parasite bam files. Finally, we combined the individual variant call format (VCF) files with CombineGVCFs and conducted joint genotyping with GenotypeGVCFs in GATK (Genomic Analysis Toolkit) for hosts and parasites separately. We filtered and subset our VCF files and counted variants with bcftools v.1.17 [32] and custom awk code. We calculated percentage GC content in the reads with seqkit v.0.11.0 [35] and examined the number of sites shared between mapped samples using samtools, custom awk code, and bedtools v.2.30.0 [36]. We parallelized parts of our code with GNU Parallel v.20230122 [37] and summarized the results with multiqc v.1.14 [38].

### Statistical analysis

The depth of coverage between samples over 10-kb windows of the genomes (host and parasite genomes separately) were correlated using multiBamSummary in deeptools v.3.5.5 [39]. We conducted a principal components analysis (PCA) in plink v.1.90 [40]. We conducted the PCA on the filtered VCF files after removing linked variants in plink (10-kb window size, 10-bp step size and *r*^2^ > 0.2 cutoff). All plotting and data formatting were conducted with R packages collected in tidyverse v.2.0.0 [41], and the R packages lme4 [42] and lmerTest [43] were used for linear mixed effects modeling. We used the Kenward-Roger approximation to the denominator degrees of freedom to generate *p* values for *F* tests in the linear mixed effects models. Finally, we conducted Pearson’s correlations in R using R v.4.3.2 [44].

## Results

### Improved parasite sequences as a result of SWGA

**Fig. 1:**
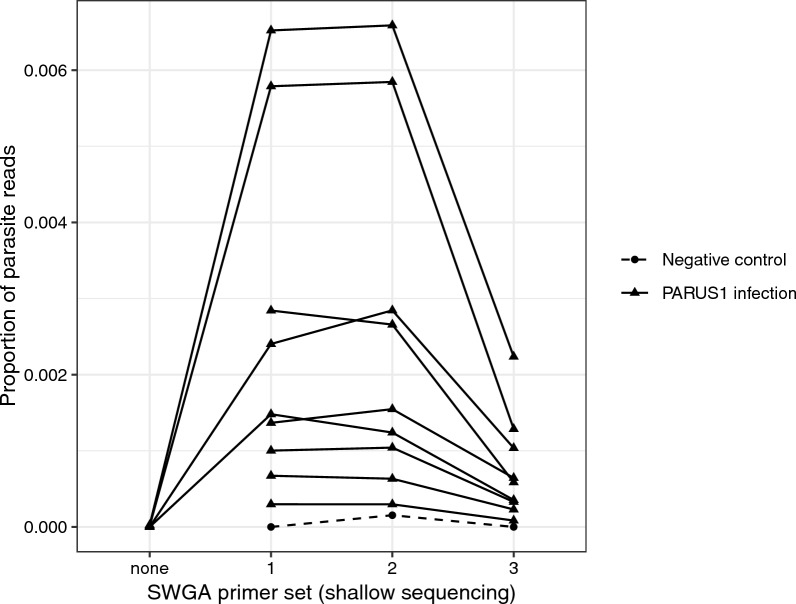
The proportion of reads that mapped to the *Haemoproteus majoris* parasite genome in unamplified (SWGA primer set “none”) DNA extracted from *H. majoris* (PARUS1 lineage)-infected blue tits (*Cyanistes caeruleus*) and DNA that underwent SWGA with one of three primer sets (1–3). Triangles denote individual samples, and the same samples sequenced under different conditions are connected with a solid line. Three negative controls (water) were amplified in each of the three SWGA primer set reactions (1 reaction per primer set) and are represented as filled circles connected with a dashed line. SWGA, Selective whole genome amplification

The four unamplified samples were sequenced to an average (± standard error [SE]) depth (i.e. total number of reads) of 73,614 ± 5047 reads (forward and reverse); of those, 72,243 ± 4849 reads passed quality filtering and were used for mapping. Nearly all reads were successfully mapped to the blue tit genome (approx. 99.9%), with only two reads from one unamplified sample, one read from another sample and none from the remaining two samples mapped to the parasite genome. SWGA increased the proportion of reads mapping to the parasite genome in all four samples (Fig. 1). For example, the unamplified sample with two parasite-mapping reads out of 80,839 total reads (2.5 × 10^–5^ or 0.0025%), increased to approximately 0.65% of reads mapping to the parasite genome with primer sets 1 and 2 on the shallow sequencing run. The shallow sequencing run of the amplified samples was sequenced to a depth (± SE) of 365,104 ± 11,496 reads (excluding negative controls which were sequenced to a depth of 13,900 ± 4484 reads).

### Primer sets

**Fig. 2:**
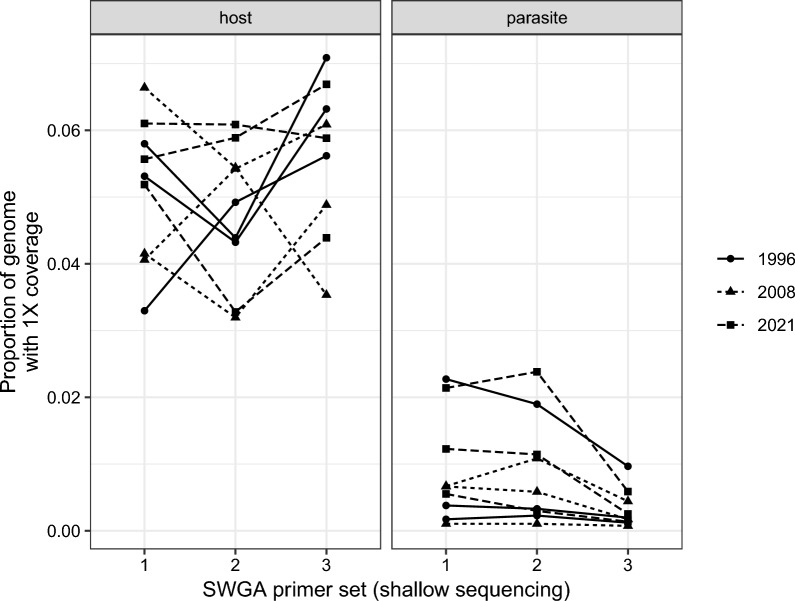
Breadth of coverage measured as the proportion of the host or parasite genomes with at least 1X depth of coverage by SWGA primer set and sample year. Breadth of coverage is positively correlated with mean read depth (*r* > 0.99), and is higher for mapping to the host genome than to the parasite genome. SWGA primer sets 1 and 2 had higher breadth of coverage (and mean depth of coverage) than primer set 3. SWGA, Selective whole genome amplification

Primer sets 1 and 2 produced a higher proportion of reads mapping to the parasite genome than primer set 3 in the shallow sequencing run (Fig. 1). Mean read depth was positively correlated with breadth of coverage, the latter measured as the proportion of the genome with at least 1X coverage (*r* > 0.99 for all host and primer combinations). Mean read depth and breadth of coverage were higher for mapping to the host genome than the parasite genome in all cases (Fig. 2). For parasite mapping, primer sets 1 and 2 had greater breadth of coverage than primer set 3 (*F*_2,16_ = 15.58, *p* < 0.001), and this was independent of sample year (i.e. age of the sample; *F*_2,6_ = 0.47, *p* = 0.646; Fig. 2).

The negative control for primer set 1 had 906 reads that passed quality filtering, of which 189 mapped to the blue tit and none mapped to the parasite. The negative control for primer set 2 had 32,478 reads, of which 13,841 mapped to the blue tit and five mapped to the parasite genomes. Finally, the negative control of primer set 3 had 48,152 reads, of which eight mapped to the blue tit genome and none to the parasite genome. Consistent with these relatively small numbers of mapped reads, we did not find bands in the agarose gels for any of the three negative controls, suggesting that there was no contamination during the SWGA reactions.

### Deep sequencing with primer set 2

**Fig. 3:**
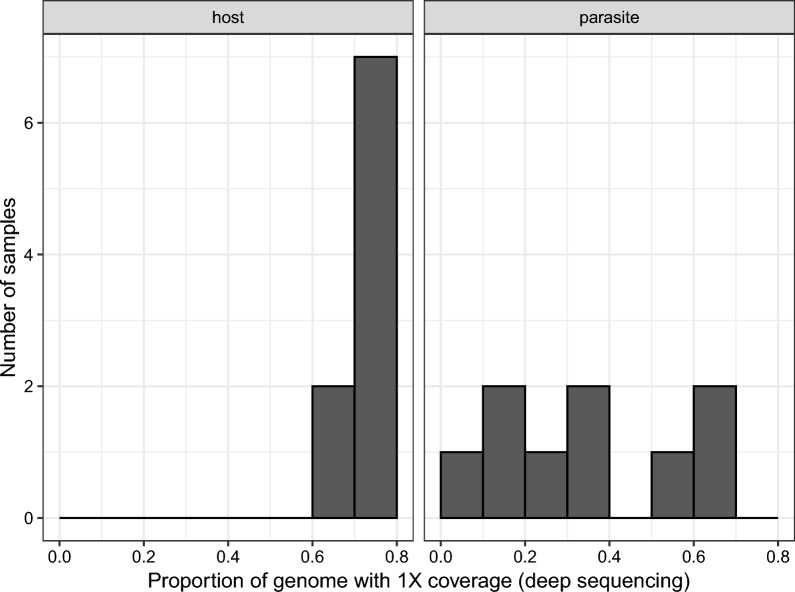
Breadth of coverage measured as the proportion of the host or parasite genomes with at least 1X depth of coverage after deep sequencing of samples amplified by SWGA. Most samples had high breadth of coverage across the host genome; variation among samples was greater for the parasite genome, as would be expected given that there is variation in parasitemia. SWGA, Selective whole genome amplification

We chose primer set 2 for deep sequencing as it seemed to work as well as primer set 1 and better than primer set 3 (Fig. 1). We re-sequenced the libraries of the nine samples prepared with SWGA primer set 2 on a NextSeq 2000 (56,426,167 ± 6,373,964 [SE] reads per sample) resulting in 53,238,047 ± 15,405,467 reads per sample that passed quality filtering. The mean (± SE) depth of coverage was 11.3 ± 1.85 for the host genome and 1.17 ± 0.446 for the parasite genome per sample. The mean (± SE) breadth of coverage (i.e. proportion of the genome with at least 1X depth of coverage) was 0.743 ± 0.020 for the host genome and 0.334 ± 0.075 for the parasite genome per sample (Fig. 3). The average (± SE) proportion of reads mapping to the parasite genome decreased slightly from 0.0025 ± 0.0008 in the shallow sequencing run to 0.0018 ± 0.0005 in the deep sequencing run (*F*_1,8_ = 36.79, *p* < 0.001).

An important aspect of any genome sequencing technique for population genomics is sequencing of the same genomic regions among samples so that genetic variants can be identified. We determined the consistency among samples by correlating the average sequencing depth of coverage over 10-kb windows between samples for the host and parasite genomes separately. The mean (± SE) correlation coefficient (*r*) among these 10-kb windows was 0.929 ± 0.007 for the host genome and 0.472 ± 0.028 for the parasite genome among samples. To understand the degree of overlap in parasite sequencing in a best-case scenario (i.e. where parasite genome sequencing works very well), we calculated the number of nucleotide positions with at least 1X depth of coverage in common between the two best sequenced infections. The two best sequenced parasite infections shared 10,821,340 nucleotide positions (approx. 45% of the reference genome) with at least 1X depth of coverage. Individually, the two infections had 14,090,382 (approx. 59% of the reference genome) and 14,646,363 (approx. 61% of the reference genome) nucleotide positions sequenced to 1X depth of coverage.

We generated separate variant files for the nine host individuals and the nine parasite infections. The raw host variant file had 16,517,700 variants (14,276,887 SNPs and 2,344,956 indels; some sites are classified by the software as both SNPs and indels so their total is greater than the number of variants); on average (± SE) there was less than one host individual missing per variant (0.912 ± 0.0004), and variant depth (123.19 ± 0.031) and quality (1012.78 ± 0.486) were high. The raw parasite variant file had 872,832 variants (787,509 SNPs and 86,449 indels); on average (± SE) about seven of nine parasite infections were missing per variant (6.737 ± 0.0015), and variant depth (14.52 ± 0.017) and quality (436.02 ± 0.645) were lower, but still relatively high. We then restricted both variant files to variants with no more than three missing individuals (host individuals or parasite infections), a minimum depth of five and a minimum quality of 30; this resulted in a filtered host variant file with 14,827,899 variants (12,806,876 SNPs and 2,118,287 indels) and a parasite variant file with 20,954 variants (17,192 SNPs and 3803 indels). For population genetics inference, one needs to know the number of sites that vary among individuals in the population (VCF, such as that used in the present study, typically calculates the number of sites that vary from the reference; however, the reference often does not come from the target population and in the case of our parasite it is not the same genetic lineage [reference is lineage WW2; samples are lineage PARUS1]). Therefore, we also counted the number of sites with more than one allele in the samples (i.e. sites that varied among individuals in the population) and found 14,512,339 such sites among the host individuals and 7068 among the parasite infections.

The 7068 variable sites for the parasites are conservative in the sense that we filtered the VCF file to include variants with six samples (infections), not all of which sequenced equally well (Fig. 3); restricting the filtering to parasite samples that sequenced well would likely reveal more variants. Therefore, we explored restricting the VCF file to the four best sequenced infections and varying the minimum read depth per variant from 1X to 5X and found additional variable sites (Table 1).

**Table 1:** The variant call format file was restricted to the four best sequenced infections (1HA37672, 1EP50341, 1EE56066, 1HA37673; Additional file 1: Table S1) and variants where all four infections were present with a minimum read depth of 1X, 2X, 3X, 4X or 5X, and minimum variant quality of 30

Minimum variant read depth	Variable sites (*n*)
1X	13,358
2X	13,276
3X	12,639
4X	11,373
5X	9895

For each of these variations in read depth, we present the number of sites that varied among infections (i.e. the number of sites with > 1 genotype)

**Fig. 4:**
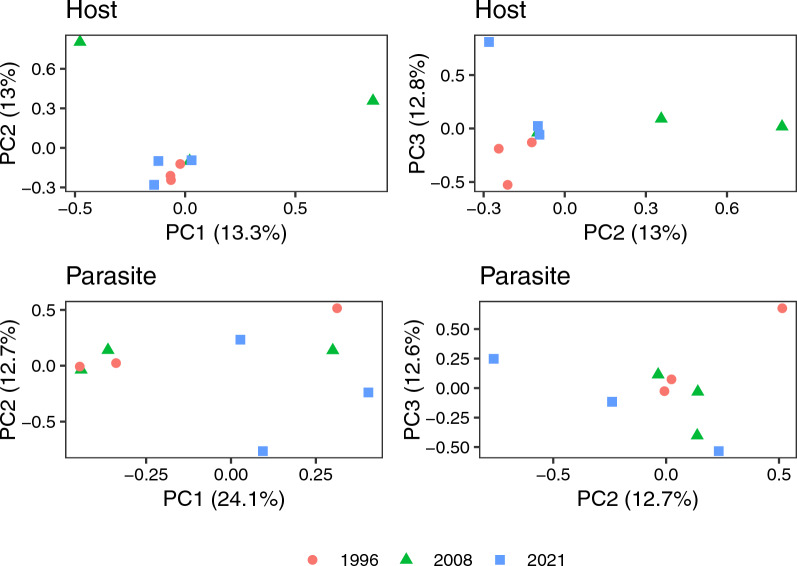
Principal components analysis of host (top two panels) and parasite (bottom two panels), with the shape and color of each individual sample corresponding to the year the sample was collected. The left side panels show the first two PCs while the right panels show the second and third PCs. All axes show the percentage of the genetic variation each PC explained in the analysis. PC, Principal component

Using the larger filtered VCF file (14,512,339 and 7068 variable sites for the hosts and parasites, respectively), we found variation among the samples. A PCA of the filtered host variants captured 39.05% of the cumulative genetic variation over the first three principal components, while a PCA of the filtered parasite variants captured 49.3% of the variation over the first three principal components; both hosts and parasite genetic diversity revealed some degree of clustering over the three time periods investigated (Fig. 4).

### Unmapped reads

**Fig. 5:**
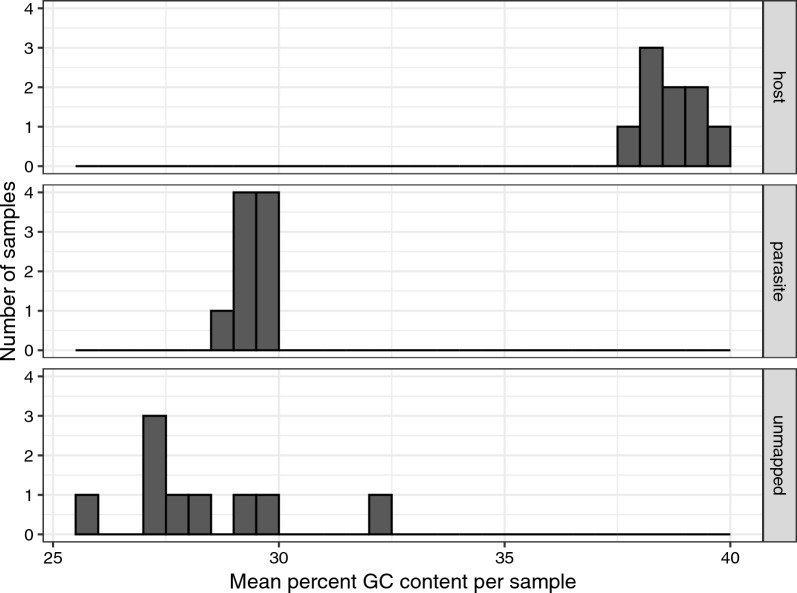
A histogram of the percentage GC content of reads that mapped to the host or the parasite, or were unmapped is presented for each sample. Parasite reads had much lower GC content than host reads and unmapped reads were more similar in GC content to the parasite reads than to the host reads

Because the reference was a different lineage of *H. majoris* than the samples we sequenced (reference lineage is WW2; samples are lineage PARUS1), some of the unmapped reads may have been parasite reads that did not map because of a high degree of divergence relative to the reference (particularly intronic regions). To explore this possibility, we examined the mean GC content of reads that mapped to the bird, the parasite and remaining unmapped reads for each of the deep sequenced samples. We found unmapped reads to have much lower GC content than bird mapped reads, and similar GC to the parasite mapped reads, suggesting that they may contain unmapped parasite reads (Fig. 5). In the deep sequenced samples, the proportion of non-host reads that did not map to the parasite (i.e. plausibly including parasite reads that were too divergent to map to the parasite reference genome) was on average (± SE) 0.549 ± 0.051 (Additional file 1: Table S2), which is a substantial proportion.

### Sequencing success and parasitemia

We quantified parasitemia (number of infected red blood cells after examining approximately 10,000 red blood cells) for five infected birds (Additional file 1: Table S1). Despite a low sample size, parasitemia was strongly positively correlated with both the proportion of the reference genome over which the infection was sequenced to at least 1X coverage (*r* = 0.99, *t* = 12.04,* df* = 3, *p* = 0.001) and mean depth of coverage (*r* = 0.96, *t* = 6.12,* df* = 3, *p* = 0.009).

## Discussion

We identified SWGA primer sets that successfully amplify the avian haemosporidian parasite *H. majoris* (lineage PARUS1) to levels that allow for whole genome sequencing of the parasite in the presence of a high concentration of host DNA (Figs. 1–3). In addition, sequencing of amplified samples allowed for dual host–parasite genome sequencing and population genetic analysis (Fig. 4). The success of SWGA is positively related to parasitemia, with higher parasitemia samples resulting in a higher proportion of parasite reads, similar to other techniques, such as sequence capture [16, 17]. Notably, samples collected 25 years apart worked equally well. While more data would facilitate a formal statistical comparison, host age and sex also do not appear to affect sequencing results (Additional file 1: Tables S1, S2). Our initial population genetic analysis revealed some degree of genetic structure among years, which was more notable for the host than for the parasite (Fig. 4). While additional samples are needed to conduct additional analyses and draw firm conclusions, this study demonstrates the utility of SWGA in avian haemosporidian population genetic and host–parasite coevolutionary studies.

**Fig. 6:**
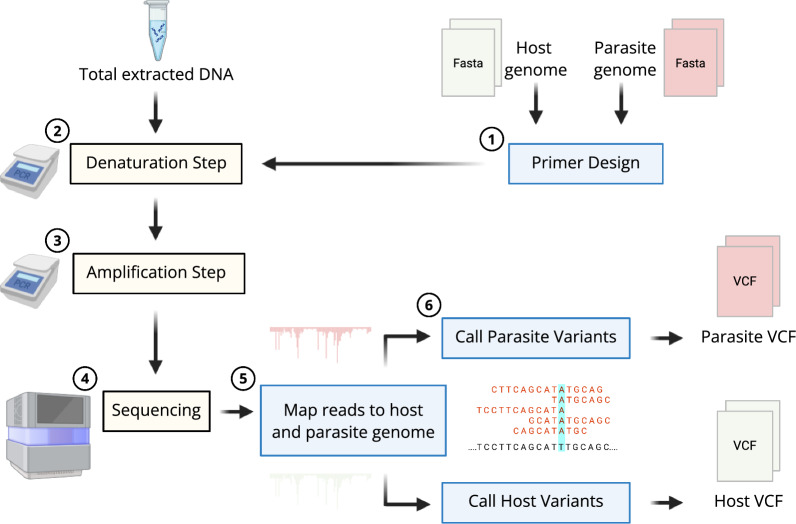
An overview of the SWGA workflow. Begin with primer design using the host and parasite genomes (1), then perform the SWGA reaction in the laboratory, starting with denaturation of the total extracted DNA (2), followed by the addition of the phi29 enzyme and amplification (3) and finally sequencing (4). Next, map sequenced reads to the host and parasite genomes (5) and call parasite and host variants separately (6) producing VCF files for the host and parasite. Laboratory steps are highlighted in light yellow and bioinformatic steps in light blue. Created in BioRender ([18]; https://BioRender.com/s587o1z). SWGA, Selective whole genome amplification, VCF variant call format

When attempting to sequence parasite or pathogen DNA from host cells and tissues, far more host DNA will be encountered than parasite DNA. Rather than treating host reads as an impediment to be removed, as in sequence capture in which host DNA is washed away, we demonstrate that SWGA allows for host DNA to be sequenced along with a greater proportion of amplified parasite DNA, thereby enabling good coverage of both host and parasite genomes from the same samples (Fig. 6). As we have shown, data from SWGA can be used for population genetic studies of both hosts and parasites and for investigations of genotype-by-genotype coevolutionary interactions between hosts and parasites [20]. We showed a significant amount of genetic variation among well-sequenced parasite infections from a single location over time (Table 1, Fig. 4), and we expect that even more variants are likely to be present in parasites from different populations [45].

SWGA has several other important benefits over previous methods of parasite sequence enrichment such as, for example, sequence capture and transcriptome sequencing, that present new opportunities for the field. First, amplification occurs across the genome and includes both coding and non-coding DNA, allowing for multiple types of genetic variation to be examined. Furthermore, as long as the primers bind to conserved sites in the haemosporidian genomes, one can amplify the genomes of related parasites, including those from different host species. For example, a single set of SWGA primers can amplify multiple species of avian *Plasmodium* parasites from multiple host species (V. Ellis, unpublished data). Finally, SWGA is relatively cheap to implement in terms of time (3 h reaction, similar to standard PCR) and reagents, allowing resources to be shifted to the cost of deeper sequencing and increasing the number of samples included in a study.

One limitation of SWGA is that, like similar methods [16, 17], it depends on parasitemia. Therefore, samples with higher parasitemia should be preferred when using this method. Indeed, analyzing parasite sequences from the four best sequenced infections revealed more variants than when using the full dataset with several relatively poorly sequenced infections included (see Results). Importantly, we mapped our reads from the genetic lineage PARUS1 against a reference genome of the lineage WW2. While most of our reads mapped to either the host or parasite (Additional file 1: Table S2), there was a substantial proportion of unmapped reads which had GC percentages more like parasite reads than host reads (Fig. 5). Therefore, mapping to the PARUS1 reference may capture diverged parasite reads. One could presumably generate a more accurate reference genome through de novo assembly [46] of reads from a single SWGA amplified sample. While high parasitemia is preferred, deeper sequencing of low parasitemia samples after SWGA could be explored to increase coverage of the parasite genome, particularly for valuable samples collected from the wild.

Avian haemosporidian researchers considering using SWGA in their system should be aware of a few points before starting work:

(i) We have not thoroughly examined how well a set of SWGA primers designed for one parasite species (or lineage) will amplify the DNA of another parasite species. In the preset study, SWGA primers designed for the lineage WW2 successfully amplified infections of PARUS1. And, as mentioned, a set of *Plasmodium* SWGA primers was successful in amplifying multiple parasite species (V. Ellis, unpublished data). Nevertheless, we have only begun to investigate how generalized these primer sets are. We assume that SWGA will be most effective if you can first generate a draft genome of your parasite of interest and then use that genome for primer design.
(ii) After identifying a list of top 10 or more primer sets, it is unclear what makes a primer set in that list better than another in the list. The only way to determine the effectiveness of an SWGA primer set is to try it. We recommend first amplifying a subset of samples with several primer sets, then shallow sequencing to see which primer set worked best. It is unclear whether combining primer sets is an effective strategy; we have not tested this in this system.
(iii) High parasitemia samples should work better than low parasitemia samples, so quantifying parasitemia prior to SWGA may be worthwhile.

## Conclusions

In the present study we have shown the utility of SWGA in the avian haemosporidian system. We expect that this method and the dual host–parasite genome sequencing approach we demonstrate here will accelerate investigations of host–parasite population genetics and coevolution in this and similar systems.

## Supplementary Information

**Supplementary Material 1: Table S1.** Descriptive information about each of the samples included in this study (variable definitions provided in the metadata sheet).** Table S2.** Statistics from all sequencing experiments (variable definitions provided in the metadata sheet).

## Data Availability

All raw sequence data generated from this study are available on GenBank (accession number: PRJNA1249433).
